# Drying kinetics and mathematical modeling of hot air drying of coconut coir pith

**DOI:** 10.1186/s40064-016-2387-y

**Published:** 2016-06-21

**Authors:** J. A. K. M. Fernando, A. D. U. S. Amarasinghe

**Affiliations:** Coconut Processing Research Division, Coconut Research Institute, Lunuwila, 61150 Sri Lanka; Department of Chemical and Process Engineering, Faculty of Engineering, University of Moratuwa, Moratuwa, 10400 Sri Lanka

**Keywords:** Coconut coir pith, Hot air drying, Mathematical modeling, Effective moisture diffusivity

## Abstract

Drying kinetics of coir pith was studied and the properties of compressed coir pith discs were analyzed. Coir pith particles were oven dried in the range of temperatures from 100 to 240 °C and the rehydration ability of compressed coir pith was evaluated by finding the volume expansion. The optimum drying temperature was found to be 140 °C. Hot air drying was carried out to examine the drying kinetics by allowing the coir pith particles to fluidize and circulate inside the drying chamber. Particle motion within the drying chamber closely resembled the particle motion in a flash dryer. The effective moisture diffusivity was found to increase from 1.18 × 10^−8^ to 1.37 × 10^−8^ m^2^/s with the increase of air velocity from 1.4 to 2.5 m/s respectively. Correlation analysis and residual plots were used to determine the adequacy of existing mathematical models for describing the drying behavior of coir pith. The empirical models, Wang and Singh model and Linear model, were found to be adequate for accurate prediction of drying behavior of coir pith. A new model was proposed by modifying the Wang and Singh model and considering the effect of air velocity. It gave the best correlation between observed and predicted moisture ratio with high value of coefficient of determination (R^2^) and lower values of root mean square error, reduced Chi square (χ^2^) and mean relative deviation (E%).

## Background

Coconut is the third main crop in Sri Lanka and Sri Lanka is the fourth largest producer of coconut in the world contributing to an average of 2500–2800 million nuts per year (Sri Lanka Coconut Statistics 2014). However, only about 10 % of the global production of coconuts (73,811 million nuts per year) is utilized in the coir industry. Sri Lanka is considered as a major coir fiber exporting country in the world, only second to India. Coir pith is a waste material generated from the husks of coconut (*Cocos nucifera* L.) during the extraction of coir fiber. Compressed value added coir pith products such as bales, briquettes, discs and grow bags contribute to more than 11 % of the total export earnings from coconut products in Sri Lanka (Sri Lanka Coconut Statistics 2012). Coir pith is an excellent growth medium due to the specific quality characteristics of high water retention ability due to the porosity of the cell structure and the fertility as it contains macro and micro nutrients (Abad et al. 2002). At present there is an increasing demand for coir pith as a sound alternative to sphagnum peat especially in soilless growth medium for containerized crop production.

Drying plays a major role in manufacturing of coir pith products. It is an important unit operation where phenomenon of heat and mass transfer occurs in between the product and the drying air (Ozdemir and Devres 1999). Reduction of moisture to a desired level is highly essential to conserve the quality of coir pith. Water retention capacity and volume expansion can be identified as the most important quality parameters required for using coir pith as a growth medium in Agricultural and Horticultural industries (Abad et al. 2002; Sarma 2008).

Sun drying under natural convection is widely used as the conventional method of coir pith drying. It is a low cost heating source (Domaz and Ismail 2011) but having some inherent disadvantages (Kooli et al. 2007). Slowness of the process, weather uncertainties specially long rainy seasons, high man power costs, large area requirement, insects infestation and contamination with foreign materials are prominent draw backs of sun drying. Sri Lanka is a tropical country and uses sun drying heavily for reduction of moisture in agricultural materials such as coir pith. At present, coir industry is not in a position to meet the current demand due to the problems associated with sun drying and hence finding alternative drying techniques is a timely need for sustaining the coir pith industry.

Hot air is used in many industrial drying applications due to several advantages including fast and uniform drying (Minguez-Mosquera et al. 1994; Ayensu 1997). Some of the coir pith manufacturers in Sri Lanka also use hot air to dry coir pith by the method of flash drying. However, they find it difficult to control the process and also to obtain required quality parameters, specifically the volume expansion. This may be mainly attributed to the lack of reliable information on suitable drying temperature and drying kinetics of coir pith during hot air drying.

Mathematical modeling and simulation of the drying curve direct better control of drying and to obtain high quality product (Meisami-asl et al. 2009). It can be further used to study the drying variables, evaluate the drying kinetics and to optimize the drying parameters and the conditions (Karathanos and Belessiotis 1999; Yun et al. 2013). The principal of modeling is based on having a set of mathematical equations which can satisfactorily describe the drying behavior (Garavand et al. 2011). Drying kinetics of agricultural materials such as tea (Raveendran et al. 2013), paddy (Manikantan et al. 2014), bird’s eye chilies (Limpaiboon 2015), jackfruit (Kaushal and Sharma 2014), alfalfa (Farhang et al. 2010), beetroot leaves (Kakade and Hathan 2014), unripe plantain chips (Famurewa and Adejumo 2015) and bay leaves (Demir et al. 2004), were successfully described by the thin layer drying models.

Coir pith is a porous agricultural material, obtaining as by product of coir fiber industry. Improper drying causes several negative effects on the structure of this biological material and the main problems can be identified as loss of rehydration ability, case hardening, color changes, shrinkage of cells, etc. Since the drying temperature is a critical parameter, this study aims at finding the most suitable drying temperature for coir pith drying. The other important parameter for drying techniques such as flash drying is the hot air flow rate and hence the main objective is to examine the effect of hot air velocity on drying kinetics of coir pith. The present study also aims at examining the applicability of existing mathematical models and to propose a new mathematical model for describing the drying kinetics of coir pith.

## Methods

### Materials

Fresh coir pith samples were collected from coir fiber mill located at Nattandiya area in Sri Lanka. Representative samples of 15 kg each were collected from the heap of coir pith at six different locations. Four were collected from the edges of the two diagonal lines perpendicular to each other, one from the top of the coir pith heap and one from the middle base of the heap. All the samples were mixed well and sieved using a half inch wire mesh to remove coir fiber.

### Laboratory dryer

**Fig. 1:**
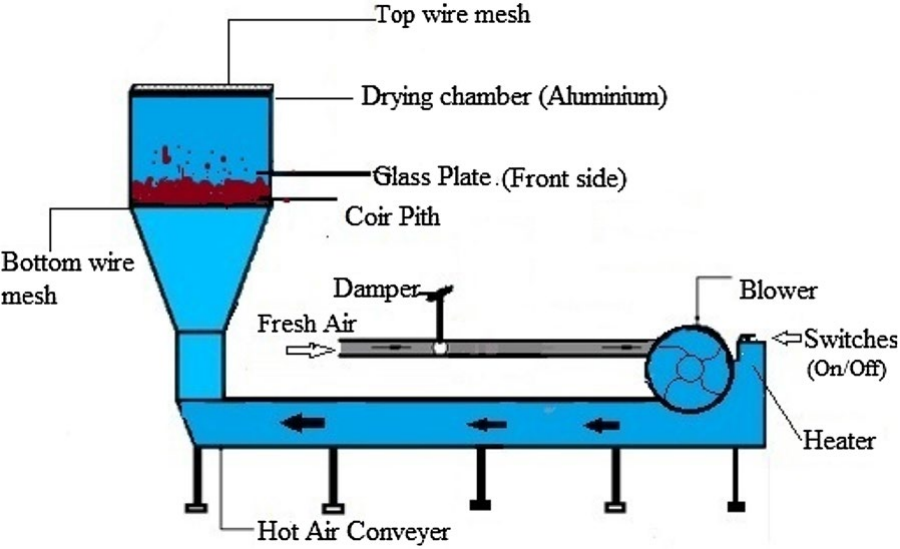
A schematic diagram of hot air dryer

Experiments were performed in a laboratory scale hot air dryer equipped with heater, blower and damper. The temperature of the hot air could be controlled with an accuracy of ±1 °C. A schematic diagram of the hot air dryer is given in Fig. 1. A wire mesh of 0.5 mm was placed at the bottom of the drying chamber to retain the coir pith while allowing the hot air to easily pass through. Another wire mess was fixed at the top to prevent elutriation of fine particles due to flow of hot air. The glass plate in front was useful to observe the fluidizing and circulating effect of coir pith. Different air velocities were obtained by regulating the damper level and thereby controlling the air flow to the drying chamber. Velocity was measured using an Anemometer (Lutron, LM 8100, Taiwan).

### Finding the optimum drying temperature

Coir pith samples of 700 ± 5 g each were oven dried at temperatures in between 100 and 240 °C at intervals of 20 °C. In industrial practice, the final moisture content of coir pith before compaction was found to be 15 ± 5 % (w/w, dry basis). A preliminary investigation was carried out to find the suitable range of final moisture content based on the volumetric expansion (VE) of the compacted discs. The highest volume expansion was recorded with the coir pith samples having final moisture content in the range of 12–23 % (w/w, dry basis). Therefore a final moisture content of 17 ± 1 % (w/w, dry basis) was selected and the drying time for each experiment was recorded. The dried samples were then compressed under 1500 psi using a hydraulic press, into the form of discs. The volume expansion of these compacted discs was measured in order to determine the optimum drying temperature.

### Drying experiments

Drying experiments were performed to determine the effect of velocity on drying of coir pith at constant temperature. Four different air flow rates corresponding to average velocities of 2.5, 2.0, 1.7 and 1.4 m/s with an accuracy of ±0.1 m/s at constant temperature of 140 ± 1 °C were used for drying coir pith samples of 300 ± 1 g each to achieve the final moisture content of 16–17 % (w/w, dry basis). The air velocities were found to be above the minimum fluidization velocity of majority of coir pith particles and hence both fluidization and circulation within the drying chamber could be observed. This drying behavior is fairly similar to the flash drying of coir pith where coir pith particles are moved with the hot air.

The initial moisture contents of fresh coir pith samples were found to be 488 ± 1 % (w/w, dry basis). All the experiments were conducted with four replicates. Weight loss of the samples during drying was measured using an electronic balance (DENVER, TP 3002, Germany) with an accuracy of ±0.01 g. The drying characteristic of coir pith was examined using the drying curves and the instantaneous drying rate, DR (g water/g dry matter per min), was calculated as

1 $$DR = \frac{{M_{t + dt} - M_{t} }}{dt}$$

where *M*_*t*+*dt*_ and *M*_*t*_ are moisture contents (g water/g dry matter) at time (*t* + *dt*) and time *t*, respectively.

### Mathematical modeling for thin layer drying

Thin layer drying models have been found wide application in describing most of the agricultural products due to their ease of use and lack of required data in complex theoretical models. Thin layer drying models fall into three categories namely, theoretical, semi-theoretical and empirical (Ozdemir and Devres 1999). The most widely investigated theoretical drying model has been Fick’s second law of diffusion. Semi-theoretical models were generally derived by simplifying general series solutions of Fick’s second law or modification of simplified diffusion models. These models describe the variation of the non-dimensional parameter *MR* (Eq. 2) with time. Among the semi-theoretical models, Newton model (Eq. 3), Page model (Eq. 4), modified Page model (Eq. 5) and Diffusion approximation model (Eq. 6) were widely used in describing the thin layer drying of food and agricultural materials (Botheju et al. 2011; Panchariya et al. 2002; Tiris et al. 1994; Kaushal and Sharma 2014). Empirical models derive a direct relationship between moisture ratio and drying time. They neglect fundamentals of the drying process and hence their parameters have no physical meaning and their applications are specific to the drying conditions of the experiments. However, due to the complex nature of moisture diffusion within the food and agricultural products empirical models are still widely used in describing the drying characteristic of these materials. Among them, Wang and Singh model (Eq. 7) has been found in the many applications such as drying of alfalfa (Farhang et al. 2010), mango pulp (Wilson et al. 2012) and sweet potato slices (Zhu and Jiang 2014). The equations derived by generalizing Fick’s second law failed to describe the drying behavior of coir pith. This may be mainly attributed to the combined effect of circulation and fluidization of coir pith particles in the drying chamber. The empirical models, Wang and Singh model and Linear model (Eq. 8), were found to give good correlation. A new empirical model was proposed (Eq. 9) by modifying the Wang and Singh model and validated. Thin layer drying models only describe the relationship between moisture ratio and drying time and hence they have limited use in modeling the applications such as flash drying where air velocity has significant effect on the drying characteristic of the material. Therefore the effect of velocity was also considered in developing the proposed model and the model parameters were estimated with the variation of velocity.

2 $$MR = \frac{{M_{t} - M_{e} }}{{M_{o} - M_{e} }}$$

3 $$MR = exp\left( { - kt} \right)$$

4 $$MR = exp\left( { - kt^{n} } \right)$$

5 $$MR = exp\left[ { - \left( {kt} \right)^{n} } \right]$$

6 $$MR = aexp\left( { - kt} \right) + \left( {1 - a} \right)exp\left( { - kbt} \right)$$

7 $$MR = 1 + at + bt^{2}$$

8 $$MR = a + bt$$

9 $$MR = \left( {1 + at + bt^{2} } \right)/(1 + ct)$$

where *MR* is the moisture ratio, *t* is the time and, *k, a, b, c* and *n* are constants; *M*_*t*_ is the moisture content at time *t* (kg water/kg dry matter); *M*_*o*_ is the initial moisture content (kg water/kg dry matter); M_e_ is the equilibrium moisture content (kg water/kg dry matter).

### Estimation of effective moisture diffusivity

The drying processes are governed by internal mass transfer resistance. Fick’s second law for diffusion can be used for the determination of drying characteristics of biological materials in the falling rate period (Arslan and Ozcan 2010; Maskan et al. 2002). The general series solution of Fick’s second law for spherical co-ordinates under the assumption of constant moisture diffusivity and temperature, and also the negligible shrinkage given by Eq. (10) (Akpinar 2006)

10 $$\frac{{M - M_{e} }}{{M_{0} - M_{e} }} = \frac{6}{{{{\uppi }}^{2} }}\mathop \sum \limits_{i = 1}^{\infty} \frac{1}{{{\text{i}}^{2} }}{ \exp }\left( { - \frac{{{\text{i}}^{2} {{\uppi }}^{2} {\text{D}}_{\text{eff}} {\text{t}}}}{{{\text{r}}^{2} }}} \right)$$

where D_eff_ is the effective moisture diffusivity (m^2^/s) and *r* is the radius of sphere.

For relatively longer drying time, Eq. (10) can be simplified as

11 $$ln\left( {\frac{{M - M_{e} }}{{M_{0} - M_{e} }}} \right) = ln\left( {\frac{6}{{{{\uppi }}^{2} }}} \right) - \left( {\frac{{{{\uppi }}^{2} {\text{D}}_{\text{eff}} }}{{{\text{r}}^{2} }}} \right)t$$

Effective diffusivity can be calculated from the gradient of Eq. (11).

### Data analysis

Nonlinear regression analysis was performed using “Lab fit” software and the statistical parameters of coefficient of determination (R^2^), root mean square error (RMSE) and reduced Chi square (χ^2^) were compared among the mathematical models to examine the validity.

The coefficient of determination (Eq. 12) is a primary criterion used to identify the best fit. In addition to R^2^, root mean square error (Eq. 13) and reduced **χ**^2^ (Eq. 14) can also be used to determine the appropriateness of the fit (Sarasavadia et al. 1999). For quality fit, R^2^ should be close to 1 while the values of the RMSE and reduced χ^2^ should be close to zero (Togrul and Pehlivan 2002; Erenturk et al. 2004; Demir et al. 2004; Goyal et al. 2006).

12 $$R^{2} = \frac{{\mathop \sum \nolimits_{i = 1}^{N} \left( {MR_{exp,i} - MR_{pre,i} } \right)^{2} }}{{\sqrt {\left[ {\mathop \sum \nolimits_{i = 1}^{N} \left( {MR_{exp,i} - MR_{pre,i} } \right)} \right]^{2} *\left[ {\mathop \sum \nolimits_{i = 1}^{N} \left( {MR_{exp,i} - MR_{pre,i} } \right)} \right]^{2} } }}$$

13 $$RMSE = \left[ {\frac{1}{N}\mathop \sum \limits_{i = 1}^{N} \left( {MR_{pre,i} - MR_{exp,i} } \right)^{2} } \right]^{1/2}$$

14 $$\chi^{2} = \frac{{\mathop \sum \nolimits_{i = 1}^{N} \left( {MR_{exp,i} - MR_{pre,i} } \right)^{2} }}{N - n}$$

where *MR*_*exp*,*i*_ and *MR*_*pre*,*i*_ are the actual and predicted moisture ratios respectively.

N is the total number of observations and n is the number of constants in the model.

The mean relative deviation E% (Eq. 15) is an absolute value that was also used in this study to examine the goodness of the fit. It gives a clear idea of the mean divergence of the estimated data from the measured data. Value of E% smaller than 5 indicates an extremely good fit; a value between 5 and 10 represents a reasonably good fit; and a value greater than 10 shows a poor fit (Lomauro et al. 1985a, b; Gencturk et al. 1986).

15 $$E\left( \% \right) = \frac{100}{N}\mathop \sum \limits_{i = 1}^{N} \left| {\frac{Experimental\,value - Predicted\,value}{Experimental\,value}} \right|$$

Parameters of the thin layer models were estimated by the method of least squares estimation using the Lab fit curve fitting software. Lab fit uses “Levenberg–Marquardt algorithm” for the estimation of nonlinear parameters.

## Results and discussion

### Optimum drying temperature

The volume expansion of compacted discs after adding water was used as the basis for finding the optimum drying temperature. Table 1 summarizes the results of volume expansion and drying time for samples dried at different temperatures. Results clearly indicate that drying at low temperatures gave higher volume expansion but at the expense of longer drying time. Based on the statistical significance at a confidence level of p < 0.05, results of volume expansion can be categorized into two groups; drying temperature of ≥160 °C and the drying temperature of ≤140 °C. Therefore 140 °C was selected as the optimum drying temperature by considering both the high volume expansion and the low drying time.

**Table 1:** Volume expansion of coir pith dried under different drying temperatures

Temperature (°C)	Volume expansion	Drying time (min)
240	3.8 ± 0.23^a^	32
220	4.12 ± 0.11^a^	36
200	3.96 ± 0.15^a^	47
180	4.16 ± 0.21^a^	58
160	4.41 ± 0.83^a^	71
140	5.61 ± 0.25^b^	105
120	5.72 ± 0.31^b^	195
100	5.96 ± 0.22^b^	300

Means with different superscript within the same column are significantly different from each other at p < 0.05 level

### Drying characteristics

**Fig. 2:**
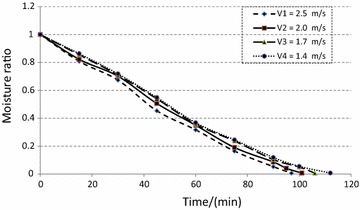
Variation of moisture ratio with time

**Fig. 3:**
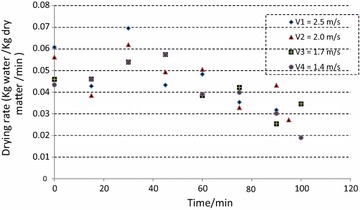
Variation of drying rate with time

Equilibrium moisture content of coir pith for the given drying conditions of 140 °C and air velocities in the range of 1.4–2.5 m/s was found to be 12.73 ± 0.65 %. At the beginning, coir pith was available as lumps and these lumps were found to break within the initial period of drying due to the fluidization and circulating effect. This behavior is relatively similar to particle motion and drying within a flash dryer. Free moisture is available in two forms; within the lumps and around the surface of particles. Figure 2 indicates that moisture content linearly reduced with time but a clear difference could be observed for the initial stage of removing free moisture and the second stage of removing the bound moisture. The drying rate corresponding to the initial period is not stable as shown in Fig. 3. This may be mainly attributed to the combined effect of removing the retained moisture in the lumps and removing of the surface moisture of coir pith after the lumps were broken. The reduction of drying rate during the falling rate period, corresponding to the removal of bound moisture, was found to be very similar to many other agricultural and food products (Famurewa and Adejumo 2015; Goyal et al.,^2007^; Togrul and Van 2003; Vega et al. 2007).

Figure 2 further indicates that a significant drop in drying time could be achieved with the increase in air velocity. The drying times corresponding to the velocities of 2.5, 2, 1.7 and 1.4 m/s were observed as 112 ± 2, 106 ± 2, 101 ± 1 and 97 ± 1 min, respectively. The increase in drying rate was attributed to the increase in convective heat transfer coefficient between the product and the air (Zhu and Jiang 2014).

### Mathematical modeling of hot air drying of coir pith with fluidization and circulation

Thin layer drying models are useful in predicting the drying kinetics of many agricultural and food materials. However, all the exponential models (Eqs. 3, 4, 5, 6) were failed to describe the drying behavior of coir pith using hot air. One of the reasons may be the circulation of coir pith particles within the drying chamber in addition to the fluidization. It was also noted that the equilibrium moisture content for coir pith was found to be 12.73 ± 0.65 % (w/w, dry basis) which is rather a higher value compared to other agricultural materials. On the other hand, the moisture content in coir pith was reduced only up to about 16–17 % in order to satisfy the secondary operation of compaction. Therefore the final stage of slow rate of drying until reaching the equilibrium moisture content was not applicable for the coir pith. However both Wang and Singh model and Linear model were found to have good correlations with the actual data. Table 2 summarizes the results of Non-linear regression analysis and the model constants for Wang and Singh model, Linear model and also the proposed new model.

**Table 2:** Model constant and statistical results for hot air drying of coir pith

Model	Velocity (m/s)	Model constants			R^2^	${\upchi }^{2} \times 10^{ - 2}$	RMSE	E%
		a	b × 10^2^	c × 10^2^				
*MR* = 1 + *at* + *bt* ^2^. (Wang and Singh model)	2.5	−0.01282	0.002552		0.9981	0.0297	0.0149	21.45
	2	−0.01174	0.001763		0.9977	0.0354	0.0175	23.95
	7	−0.01118	0.001589		0.9974	0.0430	0.0193	27.97
	1.4	−0.01120	0.001875		0.9959	0.0708	0.0248	54.84
*MR* = *a* + *bt* (Linear model)	2.5	0.96816	−1.03312		0.9931	0.1077	0.0284	82.19
	2	0.97749	−0.99589		0.9948	0.0782	0.0247	60.52
	1.7	0.98372	−0.95776		0.9938	0.0944	0.0271	69.26
	1.4	0.98068	−0.92965		0.9895	0.1585	0.0351	102.52
$MR = \frac{{\left( {1 + at + bt^{2} } \right)}}{{\left( {1 + ct} \right)}}$ (proposed model)	2.5	−0.01926	0.00925	−0.7306	0.9988	0.0221	0.0111	7.26
	2	−0.01896	0.00899	−0.7923	0.9987	0.0241	0.0126	7.77
	1.7	−0.01827	0.00836	−0.7766	0.9987	0.0249	0.0128	16.15
	1.4	−0.01799	0.00810	−0.7677	0.9986	0.0291	0.0149	5.67

According to Table 2, all three models confirm quality fit with R^2^ values close to 1 and RMSE values close to zero. However, reduced χ^2^ values for Linear model are considerably higher than the other two models. Reduced Chi square (χ^2^) is the mean square of the deviations between experimental and predicted values for the models and is a primary statistical parameter used to examine the goodness of the fit. Further, a considerable increase in χ^2^ value could be observed with the reduction of hot air velocity for the Wang and Singh model. A similar behavior was observed with the new model but the increase of χ^2^ value is only marginal. These results suggest that the new model is the most suitable model for describing the drying kinetics of coir pith using hot air with fluidization and circulation. The percent mean relative deviation modulus (E%) further confirms it with values less than 10 for three of the hot air velocities used in this study. Comparatively other two models have significantly high E% values.

### Validation of the proposed model

Model constants *a*, *b* and *c* were found to have correlation with the air velocity and they are given in Eqs. (16), (17) and (18) respectively. The numerical values of all the related constants are summarized in Table 3.

**Table 3:** Model constants for the proposed model

a		b		c	
a_1_	0.0005433	b_1_	−0.51675 × 10^−5^	c_1_	0.001268
a_2_	−0.003347	b_2_	3.13385 × 10^−5^	c_2_	−0.00465
a_3_	−0.01431	b_3_	4.67223 × 10^−5^	c_3_	−0.00361

16 $$a = a_{1} v^{2} + a_{2} v + a_{3} .$$

17 $$b = b_{1} v^{2} + b_{2} v + b_{3} .$$

18 $$a = c_{1} v^{2} + c_{2} v + c_{3} .$$

where *v* is the velocity of air.

**Fig. 4:**
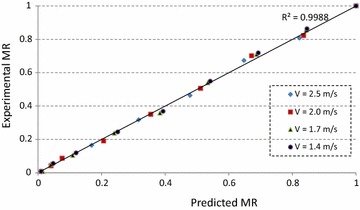
Experimental and predicted moisture ratio at different air velocities

**Fig. 5:**
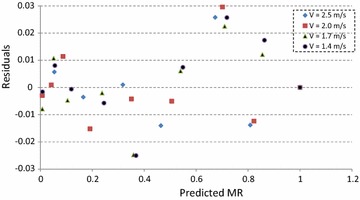
Residuals and predicted moisture ratio at different air velocities

Residual analysis was used to examine the validation of the proposed model. Figure 4 indicates that actual and predicted data have excellent correlation with R^2^ value of 0.9988. The corresponding residual plot is given in Fig. 5. Considering the overall fit, residuals are so close to the value zero and are randomly distributed, indicating the goodness of the fit (Ghaderi et al. 2012; Wisniak and Polishuk 1999).

### Effective moisture diffusivity

The effective moisture diffusivity was calculated using Eq. (11) and the values are summarized in Table 4. Moisture diffusivity has increased from 1.18 × 10^−8^ to 1.37 × 10^−8^ (m^2^/s) with the increase of air velocity from 1.4 to 2.5 m/s. Similar effect was reported in previous studies also (Arslan and Ozcan 2010; Chen et al. 2013; Velic et al. 2004; Chayjan et al. 2011; Ertekin and Yaldiz 2004).

**Table 4:** Effect of velocity on moisture diffusivity

Velocity (m/s)	Moisture diffusivity (m^2^/s)
1.4	1.18 × 10^−8^
1.7	1.22 × 10^−8^
2	1.28 × 10^−8^
2.5	1.37 × 10^−8^

## Conclusion

Volume expansion of compacted coir pith discs was found to be significantly affected by the drying temperature. An optimum temperature of 140 °C was identified for the drying of coir pith by considering both volume expansion and drying time. Effective moisture diffusivity was found to increase from 1.18 × 10^−8^ to 1.37 × 10^−8^ m^2^/s with the increase of air velocity from 1.4 to 2.5 m/s respectively. Typically used mathematical models for thin layer drying with exponential functions were failed to describe the drying behavior of coir pith. However, Wang and Singh model and Linear model gave good correlations. A new mathematical model was proposed and it was found to have the best correlation as compared to the other mathematical models used in this study. Model constants for the proposed model were found to be quadratic functions of air velocity. Since the experimental setup of this study closely simulated the particle motion and heat and mass transfer in flash drying due to induced fluidization and circulation, the new model has a great potential in designing and modeling of the flash drying of coir pith.

## List of symbols

a, b, c, k: constants in drying model
D_eff_: effective moisture diffusivity (m^2^/s)
DR: the instantaneous drying rate (g water/g dry matter per min)
E%: the mean relative deviation
M_e_: equilibrium moisture content (kg water/kg dry matter)
MR _exp, i_: actual moisture ratio
M_o_: initial moisture content (kg water/kg dry matter)
M_R_: moisture ratio
MR _pre, i_: predicted moisture ratio
M_t_: moisture content at time t (kg water/kg dry matter)
M_t+dt_: moisture content at time t + dt (kg water/kg dry matter)
n: number of constants in the model
N: number of observations
*r*: the radius of sphere
R^2^: coefficient of determination
RMSE: root mean square error
χ^2^: reduced Chi square
t: time
